# Supplementary material for: Aurora kinase A is a synthetic lethal target in FANCA-deficient cancers
Source: NPJ Precis Oncol. 2026 Jul 11;10:424. doi: 10.1038/s41698-026-01596-7 (PMC13737201; doi:10.1038/s41698-026-01596-7)
Supplement: Supplementary file 1 [file 41698_2026_1596_MOESM1_ESM.pdf]

Aurora kinase A is a synthetic lethal target in FANCA-deficient cancers

Supplementary Data legends (Excel files):

Supplementary Data 1. Prevalence of individual FA pathway gene alterations across different tumor entities. *Abbreviations: ACC: adenoid cystic carcinoma; ALL: acute lymphoblastic leukemia; CNS: central nervous system; CRC: colorectal cancer; CUP: cancer of unknown primary; DLBCL: diffuse large B-cell lymphoma; FA: Fanconi anemia; GI: gastrointestinal; GIST: gastrointestinal stromal tumor; LPD: lymphoproliferative disorders; LMS: leiomyosarcoma; MDS: myelodysplastic syndrome; MPN: myeloproliferative neoplasm; NHL: non-hodgkin lymphoma; NOS: not otherwise specified; NSCLC: non-small cell lung cancer; PCN: pancreatic cystic neoplasm; PNS: peripheral nervous system; RMS: rhabdomyosarcoma; SFT: solitary fibrous tumor; STS: soft tissue sarcoma.*

Supplementary Data 2. Molecular alteration type for individual FA pathway gene alterations across different tumor entities. *Abbreviations: ACC: adenoid cystic carcinoma; ALL: acute lymphoblastic leukemia; CNS: central nervous system; CRC: colorectal cancer; CUP: cancer of unknown primary; DLBCL: diffuse large B-cell lymphoma; FA: Fanconi anemia; GI: gastrointestinal; GIST: gastrointestinal stromal tumor; LPD: lymphoproliferative disorders; LMS: leiomyosarcoma; MDS: myelodysplastic syndrome; MPN: myeloproliferative neoplasm; NHL: non-hodgkin lymphoma; NOS: not otherwise specified; NSCLC: non-small cell lung cancer; PCN: pancreatic cystic neoplasm; PNS: peripheral nervous system; RMS: rhabdomyosarcoma; SFT: solitary fibrous tumor; STS: soft tissue sarcoma.*

Supplementary figures

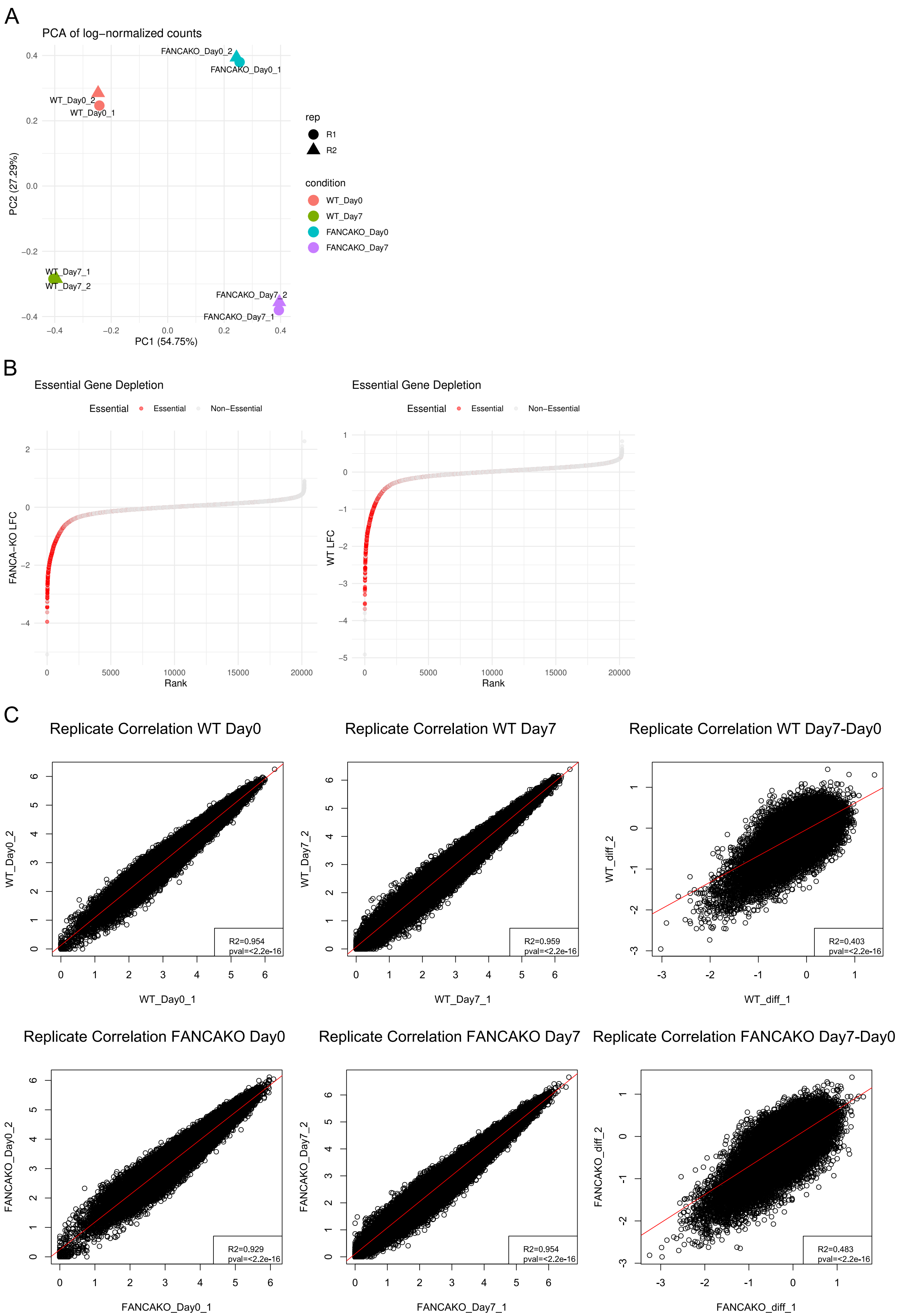
A
PCA of log−normalized counts
FANCAKO_Day0_2
FANCAKO_Day0_1
WT_Day0_2
WT_Day0_1
WT_Day7_1
WT_Day7_2
FANCAKO_Day7_2
FANCAKO_Day7_1
PC2 (27.29%)
PC1 (54.75%)
rep
R1
R2
condition
WT_Day0
WT_Day7
FANCAKO_Day0
FANCAKO_Day7
B
Essential Gene Depletion
Essential
Essential
Non−Essential
FANCA-KO LFC
WT LFC
Rank
C
Replicate Correlation WT Day0
Replicate Correlation WT Day7
Replicate Correlation WT Day7-Day0
WT_Day0_2
WT_Day0_1
R2=0.954
pval=<2.2e-16
WT_Day7_2
WT_Day7_1
R2=0.959
pval=<2.2e-16
WT_diff_2
WT_diff_1
R2=0.403
pval=<2.2e-16
Replicate Correlation FANCAKO Day0
Replicate Correlation FANCAKO Day7
Replicate Correlation FANCAKO Day7-Day0
FANCAKO_Day0_2
FANCAKO_Day0_1
R2=0.929
pval=<2.2e-16
FANCAKO_Day7_2
FANCAKO_Day7_1
R2=0.954
pval=<2.2e-16
FANCAKO_diff_2
FANCAKO_diff_1
R2=0.483
pval=<2.2e-16

Supplementary Figure 1. Quality control of the CRISPR/Cas9 synthetic lethality screen. a) PCA plot showing sample grouping and replicate correlation. b) Ranked plot of gene LFC values in MAGeCK RRA in both *FANCA*^{WT} and *FANCA*^{KO} cells, highlighting essential gene depletion as a screen quality indicator. List of essential genes was obtained from Depmap 23Q2 public (Broad Insititute, 2023). c) sgRNA count correlation plots for all CRISPR/Cas9 synthetic lethality screen samples showing good individual timepoint replicate correlation as well as the consistency of sgRNA abundance changes during the screen. *Abbreviations: diff: difference; KO: knockout; LFC: log fold change; PC: principle component; PCA: principal component analysis; pval: p value; R, rep: replicate; sgRNA: single guide RNA; WT: wild type.*

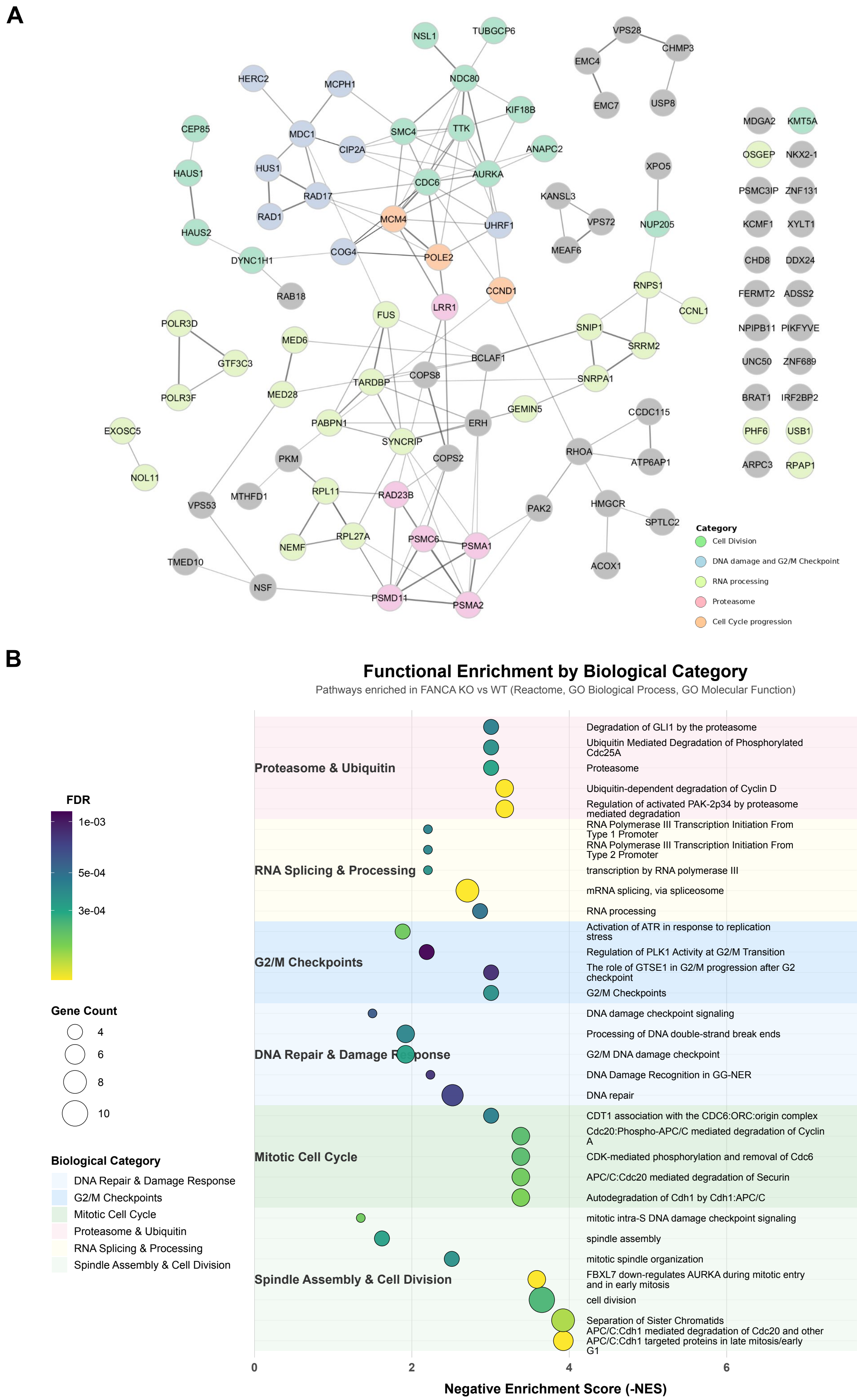
A
NSL1
TUBGCP6
VPS28
CHMP3
EMC4
HERC2
MCPH1
NDC80
EMC7
USP8
MDGA2
KMT5A
CEP85
KIF18B
MDC1
SMC4
TTK
CIP2A
ANAPC2
OSGEP
NKX2-1
HUS1
XPO5
HAUS1
AURKA
CDC6
PSMC3IP
ZNF131
RAD17
KANSL3
MCM4
RAD1
UHRF1
VPS72
NUP205
KCMF1
XYLT1
HAUS2
COG4
MEAF6
POLE2
CHD8
DDX24
DYNC1H1
RAB18
CCND1
RNPS1
FERMT2
ADSS2
LRR1
CCNL1
FUS
POLR3D
SNIP1
NPIPB11
PIKFYVE
MED6
SRRM2
GTF3C3
BCLAF1
UNC50
ZNF689
COPS8
TARDBP
SNRPA1
POLR3F
MED28
BRAT1
IRF2BP2
GEMIN5
CCDC115
PABPN1
ERH
EXOSC5
PHF6
USB1
SYNCRIP
RHOA
PKM
COPS2
ATP6AP1
ARPC3
RPAP1
NOL11
RPL11
MTHFD1
RAD23B
HMGCR
VPS53
PAK2
SPTLC2
RPL27A
NEMF
PSMC6
PSMA1
TMED10
ACOX1
NSF
PSMD11
PSMA2
Category
Cell Division
DNA damage and G2/M Checkpoint
RNA processing
Proteasome
Cell Cycle progression
B
Functional Enrichment by Biological Category
Pathways enriched in FANCA KO vs WT (Reactome, GO Biological Process, GO Molecular Function)
FDR
1e-03
5e-04
3e-04
Gene Count
4
6
8
10
Biological Category
DNA Repair & Damage Response
G2/M Checkpoints
Mitotic Cell Cycle
Proteasome & Ubiquitin
RNA Splicing & Processing
Spindle Assembly & Cell Division
Proteasome & Ubiquitin
Degradation of GLI1 by the proteasome
Ubiquitin Mediated Degradation of Phosphorylated Cdc25A
Proteasome
Ubiquitin-dependent degradation of Cyclin D
Regulation of activated PAK-2p34 by proteasome mediated degradation
RNA Splicing & Processing
RNA Polymerase III Transcription Initiation From Type 1 Promoter
RNA Polymerase III Transcription Initiation From Type 2 Promoter
transcription by RNA polymerase III
mRNA splicing, via spliceosome
RNA processing
G2/M Checkpoints
Activation of ATR in response to replication stress
Regulation of PLK1 Activity at G2/M Transition
The role of GTSE1 in G2/M progression after G2 checkpoint
G2/M Checkpoints
DNA Repair & Damage Response
DNA damage checkpoint signaling
Processing of DNA double-strand break ends
G2/M DNA damage checkpoint
DNA Damage Recognition in GG-NER
DNA repair
Mitotic Cell Cycle
CDT1 association with the CDC6:ORC:origin complex
Cdc20:Phospho-APC/C mediated degradation of Cyclin A
CDK-mediated phosphorylation and removal of Cdc6
APC/C:Cdc20 mediated degradation of Securin
Autodegradation of Cdh1 by Cdh1:APC/C
Spindle Assembly & Cell Division
mitotic intra-S DNA damage checkpoint signaling
spindle assembly
mitotic spindle organization
FBXL7 down-regulates AURKA during mitotic entry and in early mitosis
cell division
Separation of Sister Chromatids
APC/C:Cdh1 mediated degradation of Cdc20 and other APC/C:Cdh1 targeted proteins in late mitosis/early G1
0
2
4
6
Negative Enrichment Score (-NES)

Supplementary Figure 2. Pathway enrichment analysis of the hits identified in the CRISPR/Cas9 synthetic lethality screen. Whole genome loss-of-function screen was performed on isogenic FANCA -proficient vs -deficient DU145 cells. a) STRING interaction map of the hits identified in the CRISPR/Cas9 synthetic lethality screen. b) Extended pathway functional enrichment of the hits identified in the CRISPR/Cas9 synthetic lethality screen. Weighted hypergeometric analysis was performed on Gene Ontology and REACTOME databases. *Abbreviations: FDR: false discovery rate; GO: gene ontology; KO: knockout; WT: wild type.*

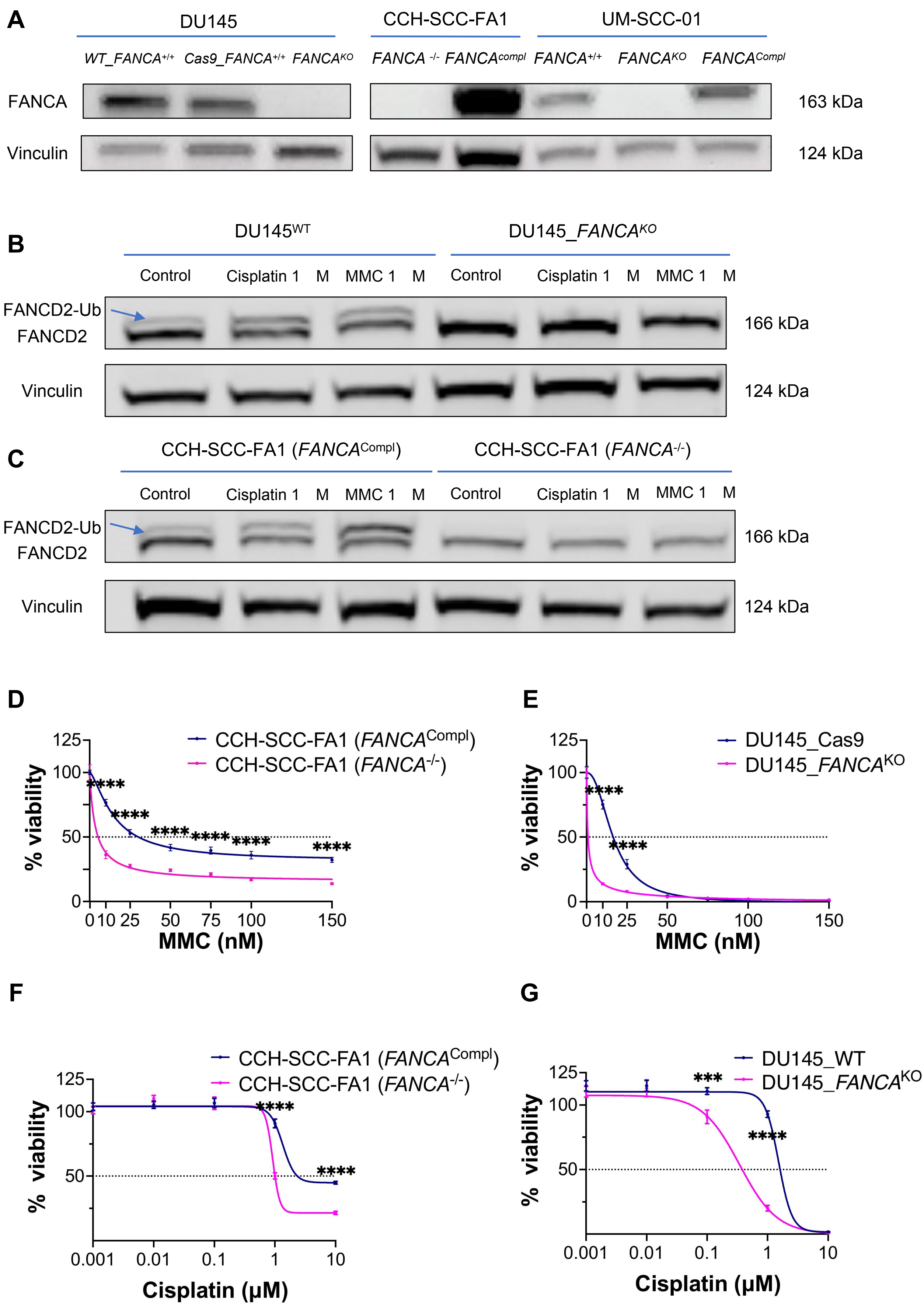

A
DU145
CCH-SCC-FA1
UM-SCC-01
WT_FANCA+/+ Cas9_FANCA+/+ FANCAKO
FANCA -/- FANCAcompl
FANCA+/+ FANCAKO FANCACompl
FANCA
Vinculin
163 kDa
124 kDa
B
DU145WT
DU145_FANCAKO
Control Cisplatin 1 M MMC 1 M Control Cisplatin 1 M MMC 1 M
FANCD2-Ub
FANCD2
Vinculin
166 kDa
124 kDa
C
CCH-SCC-FA1 (FANCACompl)
CCH-SCC-FA1 (FANCA-/-)
Control Cisplatin 1 M MMC 1 M Control Cisplatin 1 M MMC 1 M
FANCD2-Ub
FANCD2
Vinculin
166 kDa
124 kDa
D
CCH-SCC-FA1 (FANCACompl)
CCH-SCC-FA1 (FANCA-/-)
% viability
MMC (nM)
E
DU145_Cas9
DU145_FANCAKO
% viability
MMC (nM)
F
CCH-SCC-FA1 (FANCACompl)
CCH-SCC-FA1 (FANCA-/-)
% viability
Cisplatin (μM)
G
DU145_WT
DU145_FANCAKO
% viability
Cisplatin (μM)

Supplementary Figure 3. Extended data: functional characterization of FANCA-deficient vs proficient prostate adenocarcinoma and head-and-neck squamous carcinoma isogenic cell lines, including Fanconi anemia patient-derived cell lines. a) Western blot illustrating FANCA protein loss in FANCA-deficient cell lines. b-c) Lack of FANCD2 monoubiquitination, a hallmark of FA pathway activation, in FANCA-deficient cells. d-e) FANCA-deficient cells show increased vulnerability to MMC: drug response curves for CCH-SCC-FA1 (*FANCA*$^{-/-}$) vs CCH-SCC-FA1 (*FANCA*^{Compl}) (d) and DU145^{WT} vs DU145_*FANCA*^{KO} (e) are illustrated. f-g) FANCA-deficient cells show increased vulnerability to cisplatin: drug response curves for CCH-SCC-FA1 (*FANCA*$^{-/-}$) vs CCH-SCC-FA1 (*FANCA*^{Compl}) (f) and DU145^{WT} vs DU145_*FANCA*^{KO} (g) are illustrated. *Abbreviations: FANCD2-Ub: monoubiquitinated FANCD2; KO: knockout; kDa: kilodalton; μM: micromolar; nM: nanomolar.*

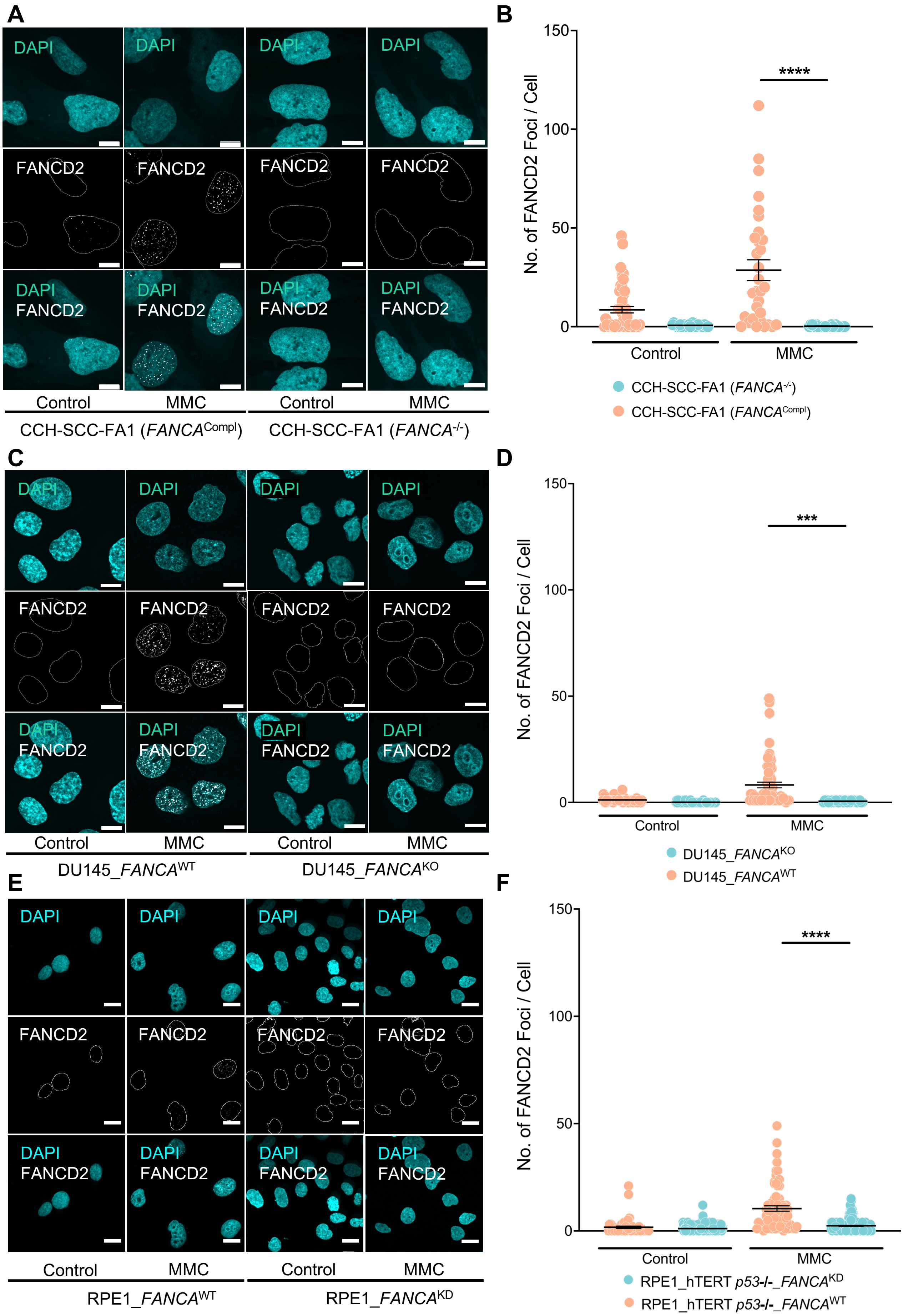
A
B
C
D
E
F
DAPI
FANCD2
DAPI FANCD2
Control
MMC
CCH-SCC-FA1 (FANCA^Compl)
CCH-SCC-FA1 (FANCA-/-)
DU145_FANCA^WT
DU145_FANCA^KO
RPE1_FANCA^WT
RPE1_FANCA^KD
No. of FANCD2 Foci / Cell
150
100
50
0
****
***
CCH-SCC-FA1 (FANCA-/-)
CCH-SCC-FA1 (FANCA^Compl)
DU145_FANCA^KO
DU145_FANCA^WT
RPE1_hTERT p53-/-_FANCA^KD
RPE1_hTERT p53-/-_FANCA^WT

Supplementary Figure 4. Extended data: FANCD2 foci formation in FANCA-deficient vs proficient isogenic cell lines under treatment with MMC and in control conditions. Cells were exposed to 1 μM MMC for 24 hours vs control conditions (DMSO) and imaged via confocal microscopy using Zeiss LSM 710 or Zeiss LSM 980 (63x). a,c,e) Representative immunofluorescence images for CCH-SCC-FA1 (*FANCA*^{Compl}) vs CCH-SCC-FA1 (*FANCA*$^{-/-}$) (a), DU145^{WT} vs DU145_*FANCA*^{KO} (c), and RPE1^{WT} vs RPE1_*FANCA*^{KD} (e); scale represents 20 μm. b, d, f) FANCD2 foci quantification for the same FANCA-deficient vs proficient cell lines. The number of foci per cell are represented, and statistical significance was calculated with the unpaired t-test. *Abbreviations: DAPI: 4',6-diamidino-2-phenylindole; KD: knockdown; KO: knockout; MMC: mitomycin C; WT: wild type.*

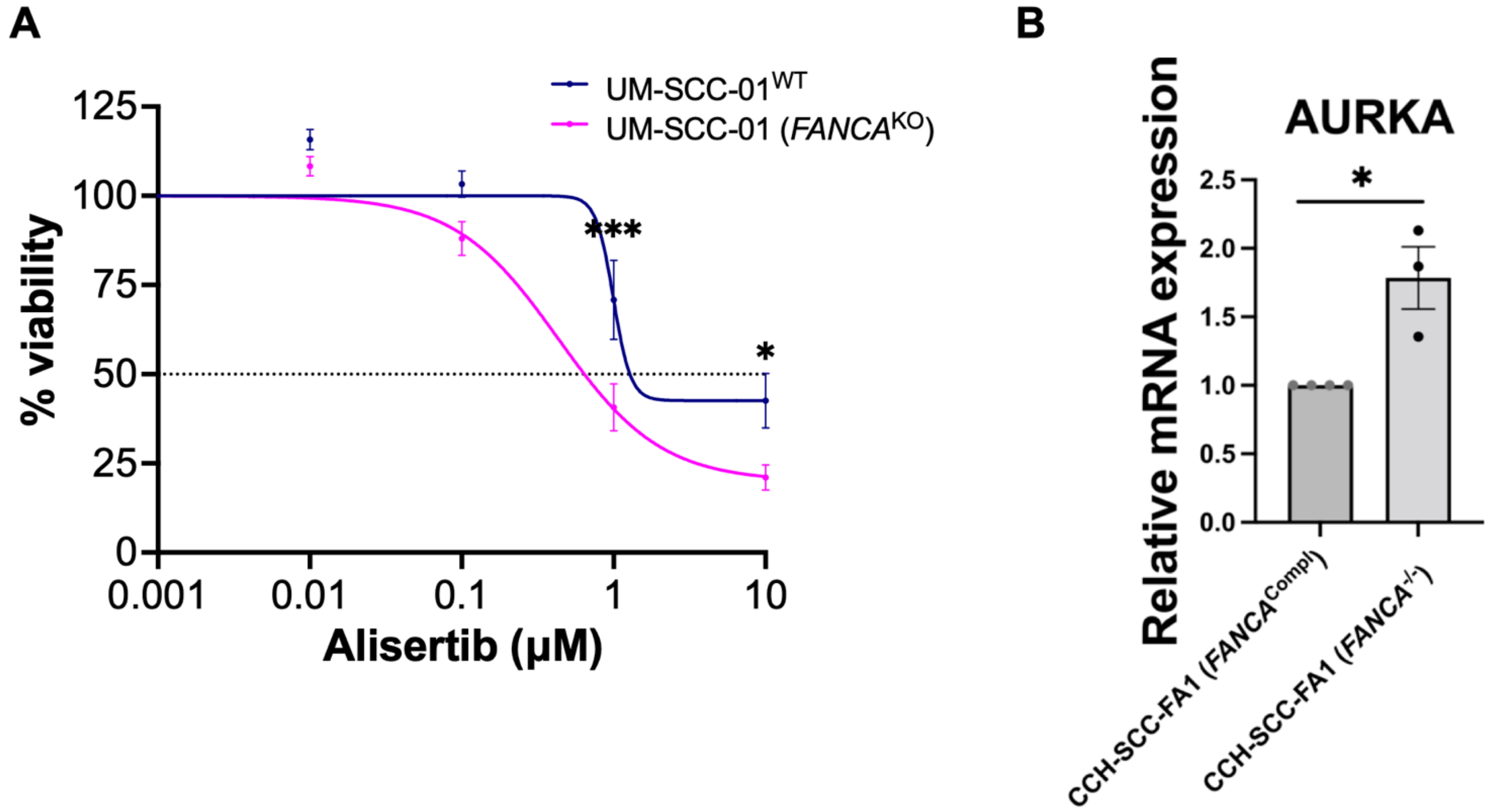

Supplementary Figure 5. Extended data: *In vitro* hit validation from CRISPR/Cas9 whole-genome synthetic lethality and high-throughput drug screens confirms that FANCA loss is synthetically lethal with AURKA inhibition *in vitro*. a) Drug response curves, assessed by CellTiter-Glo luminescence cell viability assay, illustrating increased vulnerability to the AURKA inhibitor alisertib of FANCA-deficient squamous head-and-neck cancer cell lines UM-SCC-01 (*FANCA*^{WT}), as compared to the isogenic *FANCA*^{KO} cells, UM-SCC-01 (*FANCA*$^{-/-}$). Statistical significance was calculated using the 2-way ANOVA followed by Bonferroni's multiple comparison test. b) Basal relative *AURKA* mRNA expression for the CCH-SCC-FA1 (*FANCA*$^{-/-}$) vs CCH-SCC-FA1 (*FANCA*^{Compl}) cell lines shows increased *AURKA* expression in FANCA-deficient cells. Statistical significance was calculated using the Mann-Whitney U test. *Abbreviations: AURKA: Aurora kinase A; KO: knockout; µM: micromolar; mRNA: messenger RNA; WT: wild type.*

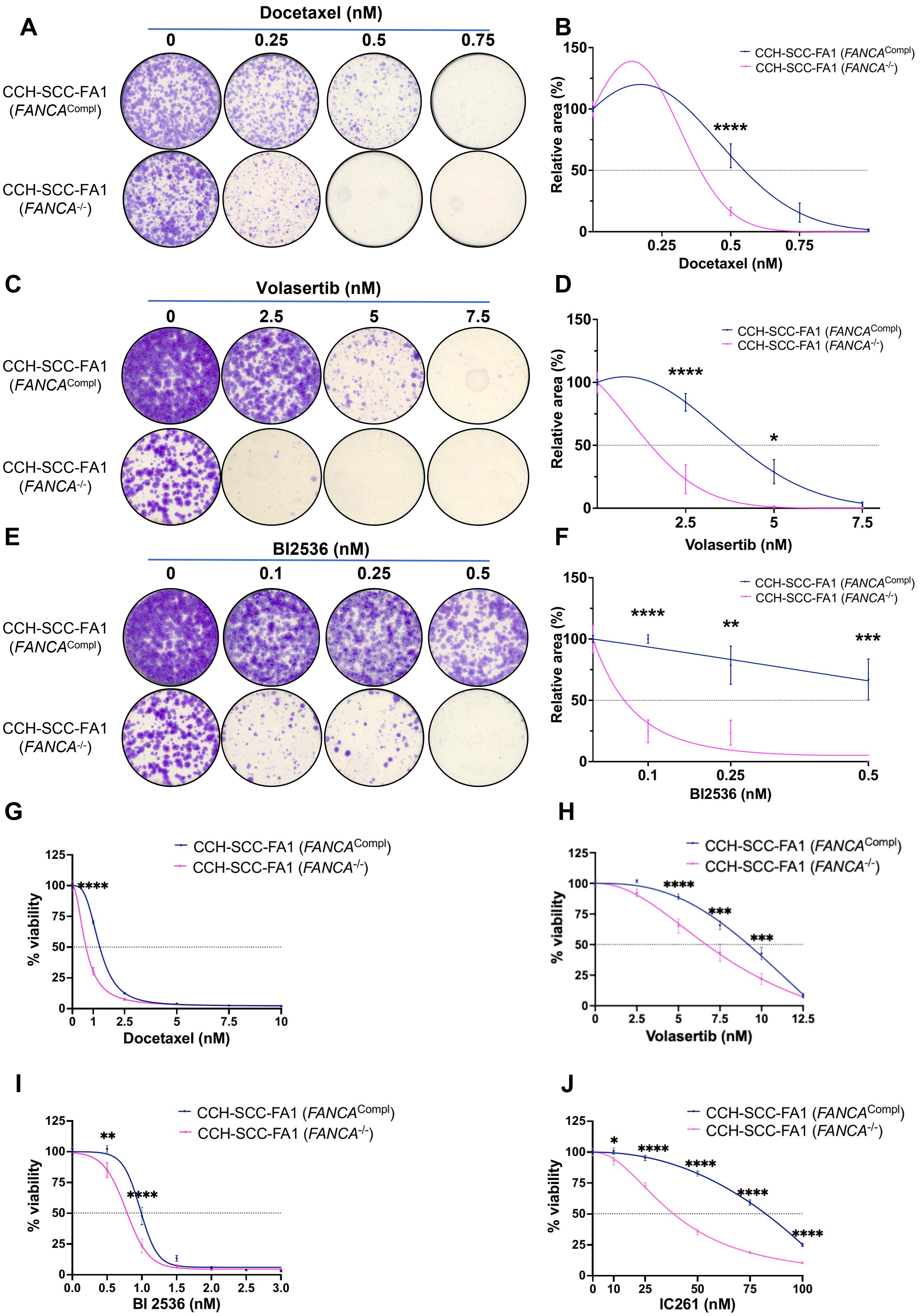
A
Docetaxel (nM)
0
0.25
0.5
0.75
CCH-SCC-FA1 (FANCA^Compl)
CCH-SCC-FA1 (FANCA-/-)
B
CCH-SCC-FA1 (FANCA^Compl)
CCH-SCC-FA1 (FANCA-/-)
Relative area (%)
Docetaxel (nM)
****
C
Volasertib (nM)
0
2.5
5
7.5
CCH-SCC-FA1 (FANCA^Compl)
CCH-SCC-FA1 (FANCA-/-)
D
Relative area (%)
Volasertib (nM)
E
BI2536 (nM)
0
0.1
0.25
0.5
CCH-SCC-FA1 (FANCA^Compl)
CCH-SCC-FA1 (FANCA-/-)
F
Relative area (%)
BI2536 (nM)
G
% viability
Docetaxel (nM)
H
% viability
Volasertib (nM)
I
% viability
BI 2536 (nM)
J
% viability
IC261 (nM)

Supplementary Figure 6. Extended data: *in vitro* hit validation from CRISPR/Cas9 whole-genome synthetic lethality and high-throughput drug screens for additional mitosis-regulating drugs. a-f). Clonogenic growth assays for FANCA-deficient vs proficient isogenic CCH-SCC-FA1 (*FANCA*$^{-/-}$) vs CCH-SCC-FA1 (*FANCA*^{Compl}) cells under treatment with docetaxel (a,b), volasertib (c,d), and BI2536 (e,f): representative images (a,c,e) and relative area (b,d,f). Statistical significance was calculated with 2-way ANOVA followed by Šidák's multiple comparison test. g-i) Drug response curves illustrating increased vulnerability to docetaxel (g), volasertib (h), BI2536 (i) and IC261 (j) of FANCA-deficient vs proficient isogenic CCH-SCC-FA1 (*FANCA*$^{-/-}$) vs CCH-SCC-FA1 (*FANCA*^{Compl}). Statistical significance was calculated using the 2-way ANOVA followed by Bonferroni's multiple comparison test. *Abbreviations: nM: nanomolar.*

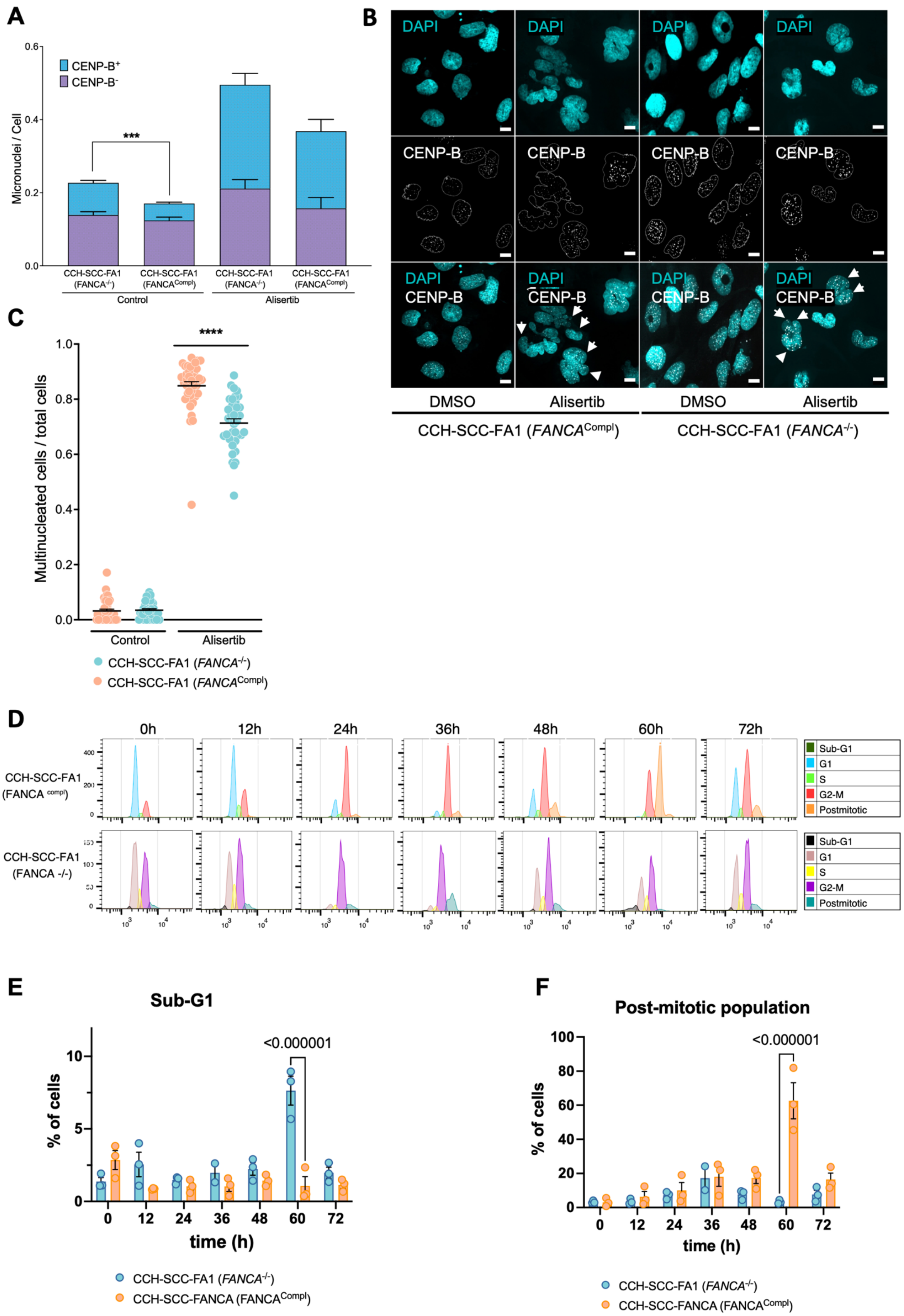
A
CENP-B+
CENP-B-
Micronuclei / Cell
***
CCH-SCC-FA1 (FANCA-/-)
CCH-SCC-FA1 (FANCACompl)
Control
Alisertib
B
DAPI
CENP-B
DMSO
Alisertib
CCH-SCC-FA1 (FANCACompl)
CCH-SCC-FA1 (FANCA-/-)
C
Multinucleated cells / total cells
****
Control
Alisertib
CCH-SCC-FA1 (FANCA-/-)
CCH-SCC-FA1 (FANCACompl)
D
0h
12h
24h
36h
48h
60h
72h
CCH-SCC-FA1 (FANCA compl)
CCH-SCC-FA1 (FANCA -/-)
Sub-G1
G1
S
G2-M
Postmitotic
E
Sub-G1
<0.000001
% of cells
time (h)
CCH-SCC-FA1 (FANCA-/-)
CCH-SCC-FANCA (FANCACompl)
F
Post-mitotic population
<0.000001
% of cells
time (h)
CCH-SCC-FA1 (FANCA-/-)
CCH-SCC-FANCA (FANCACompl)

Supplementary Figure 7. Extended data: in vitro validation of vulnerability to alisertib. a) Quantification of CENP-B foci in micronuclei under treatment with alisertib (500 nM) vs. control conditions: the number of foci per non-multinucleated cells is represented; statistical significance refers to proportion of CENP-B positive micronuclei and was calculated with the one-way ANOVA test followed by Šidák's multiple comparison test. b-c) Quantification and (b) and representative images (c) of multinucleated cells under treatment with alisertib (500 nM) vs control conditions. Treatment was performed for 48 hours. Scale represents 10 μm. Statistical significance was calculated with the one-way ANOVA test followed by Šidák's multiple comparison test. d) Representative biological replicate from cell cycle analysis under treatment with alisertib 500 nM in CCH-SCC-FA1 ($FANCA^{-/-}$) vs CCH-SCC-FA1 ($FANCA^{Compl}$) cells at 0, 12, 24, 36, 48, 60 and 72 hours. e-f) Fraction of sub-G1 (e) and post-mitotic (f) cell population in CCH-SCC-FA1 ($FANCA^{-/-}$) vs CCH-SCC-FA1 ($FANCA^{Compl}$) cells at 0, 12, 24, 36, 48, 60 and 72 hours. Statistical differences were calculated with paired t-tests followed by multiple testing correction using the FDR method. *Abbreviations: CENP-B: centromeric protein B; DAPI: 4',6-diamidino-2-phenylindole; FDR: false discovery rate; h: hours; nM: nanomolar; S: synthesis.*

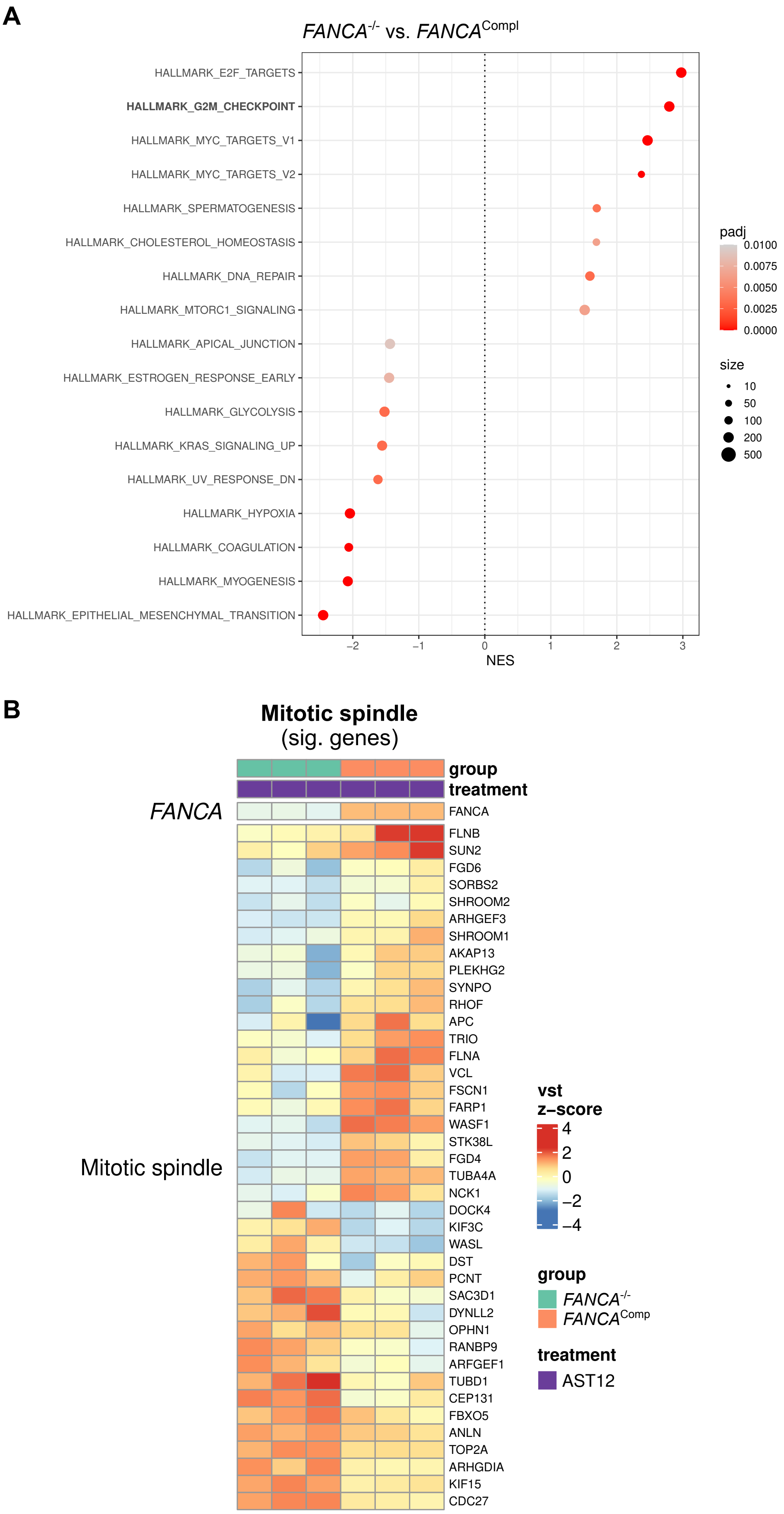
A
FANCA-/- vs. FANCACompl
HALLMARK_E2F_TARGETS
HALLMARK_G2M_CHECKPOINT
HALLMARK_MYC_TARGETS_V1
HALLMARK_MYC_TARGETS_V2
HALLMARK_SPERMATOGENESIS
HALLMARK_CHOLESTEROL_HOMEOSTASIS
HALLMARK_DNA_REPAIR
HALLMARK_MTORC1_SIGNALING
HALLMARK_APICAL_JUNCTION
HALLMARK_ESTROGEN_RESPONSE_EARLY
HALLMARK_GLYCOLYSIS
HALLMARK_KRAS_SIGNALING_UP
HALLMARK_UV_RESPONSE_DN
HALLMARK_HYPOXIA
HALLMARK_COAGULATION
HALLMARK_MYOGENESIS
HALLMARK_EPITHELIAL_MESENCHYMAL_TRANSITION
−2 −1 0 1 2 3
NES
padj
0.0100
0.0075
0.0050
0.0025
0.0000
size
10
50
100
200
500
B
Mitotic spindle
(sig. genes)
group
treatment
FANCA
FANCA
FLNB
SUN2
FGD6
SORBS2
SHROOM2
ARHGEF3
SHROOM1
AKAP13
PLEKHG2
SYNPO
RHOF
APC
TRIO
FLNA
VCL
FSCN1
FARP1
WASF1
STK38L
FGD4
TUBA4A
NCK1
DOCK4
KIF3C
WASL
DST
PCNT
SAC3D1
DYNLL2
OPHN1
RANBP9
ARFGEF1
TUBD1
CEP131
FBXO5
ANLN
TOP2A
ARHGDIA
KIF15
CDC27
Mitotic spindle
vst
z−score
4
2
0
−2
−4
group
FANCA-/-
FANCAComp
treatment
AST12

Supplementary Figure 8. Extended data: in vitro validation of vulnerability to alisertib, part 2. a) Functional enrichment analysis of bulk RNA sequencing from CCH-SCC-FA1 (*FANCA*$^{-/-}$) vs CCH-SCC-FA1 (*FANCA*^{Compl}) cells shows increased expression of the G2/M hallmark; basal levels are shown. b) Heatmap illustrating individual genes from the mitotic spindle hallmark for CCH-SCC-FA1 (*FANCA*$^{-/-}$) vs CCH_SCC_FA1 (*FANCA*^{Compl}) cells after 12-h treatment with alisertib: upregulation of centrosome-involved genes is observed for the FANCA-deficient cells (*CEP 131, KIF15, TOP2A*). *Abbreviations: AST: alisertib; FA neg.: CCH-SCC-FA1 (FANCA*$^{-/-}$*); FA pos.: CCH-SCC-FA1 (FANCA*^{Compl}*); NES: normalized enrichment score; padj: adjusted p value; VST: variance stabilizing transformation.*

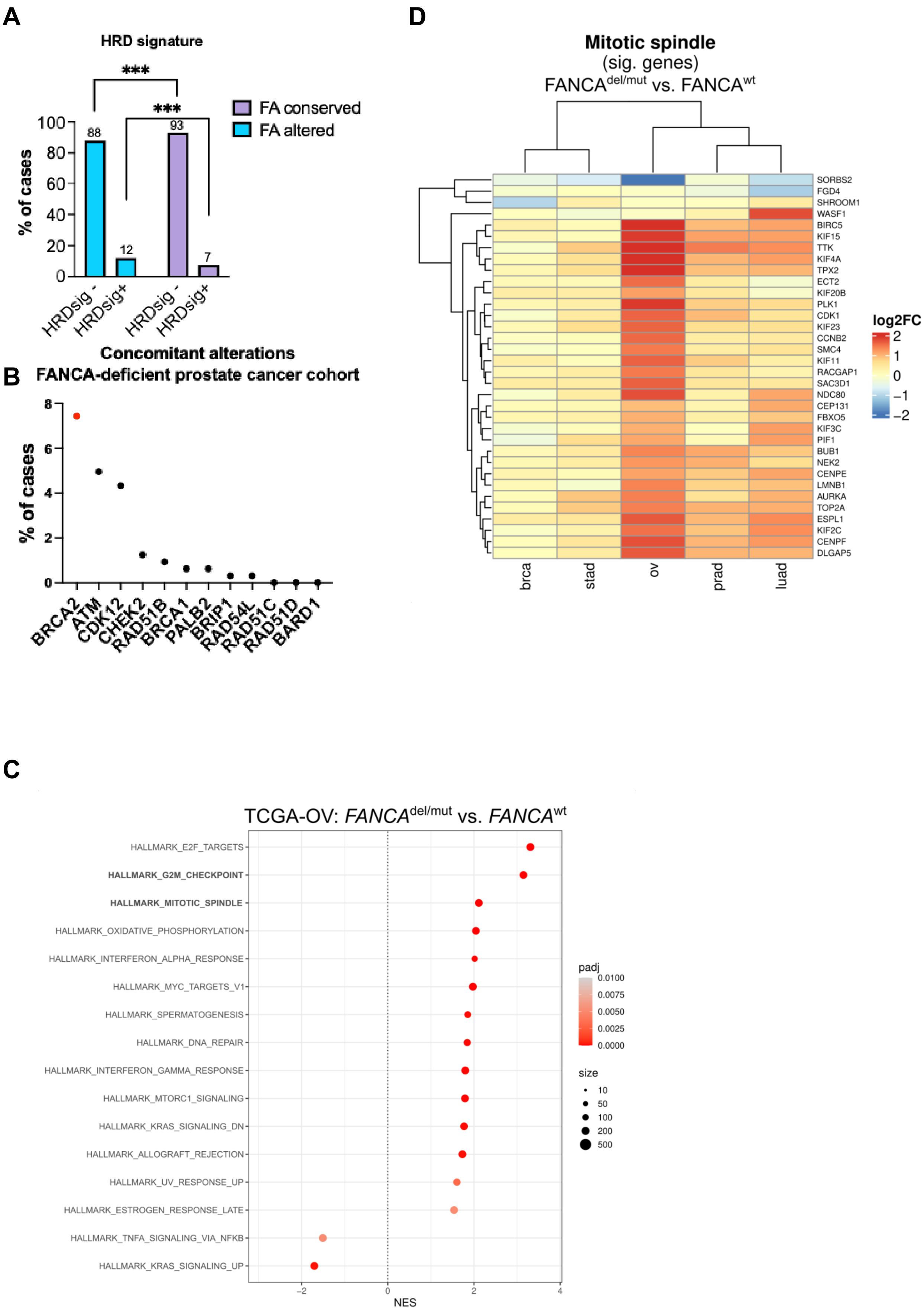
A
HRD signature
***
***
FA conserved
FA altered
% of cases
100
80
60
40
20
0
88
12
93
7
HRDsig -
HRDsig+
HRDsig -
HRDsig+
Concomitant alterations
B
FANCA-deficient prostate cancer cohort
% of cases
8
6
4
2
0
BRCA2
ATM
CDK12
CHEK2
RAD51B
BRCA1
PALB2
BRIP1
RAD54L
RAD51C
RAD51D
BARD1
C
TCGA-OV: FANCA del/mut vs. FANCA wt
HALLMARK_E2F_TARGETS
HALLMARK_G2M_CHECKPOINT
HALLMARK_MITOTIC_SPINDLE
HALLMARK_OXIDATIVE_PHOSPHORYLATION
HALLMARK_INTERFERON_ALPHA_RESPONSE
HALLMARK_MYC_TARGETS_V1
HALLMARK_SPERMATOGENESIS
HALLMARK_DNA_REPAIR
HALLMARK_INTERFERON_GAMMA_RESPONSE
HALLMARK_MTORC1_SIGNALING
HALLMARK_KRAS_SIGNALING_DN
HALLMARK_ALLOGRAFT_REJECTION
HALLMARK_UV_RESPONSE_UP
HALLMARK_ESTROGEN_RESPONSE_LATE
HALLMARK_TNFA_SIGNALING_VIA_NFKB
HALLMARK_KRAS_SIGNALING_UP
−2
0
2
4
NES
padj
0.0100
0.0075
0.0050
0.0025
0.0000
size
10
50
100
200
500
D
Mitotic spindle
(sig. genes)
FANCA del/mut vs. FANCA wt
SORBS2
FGD4
SHROOM1
WASF1
BIRC5
KIF15
TTK
KIF4A
TPX2
ECT2
KIF20B
PLK1
CDK1
KIF23
CCNB2
SMC4
KIF11
RACGAP1
SAC3D1
NDC80
CEP131
FBXO5
KIF3C
PIF1
BUB1
NEK2
CENPE
LMNB1
AURKA
TOP2A
ESPL1
KIF2C
CENPF
DLGAP5
log2FC
2
1
0
−1
−2
brca
stad
ov
prad
luad

Supplementary Figure 9. Extended data: genomic landscape of Fanconi anemia pathway gene alterations in human cancer. a) Frequency of tumor with negative vs positive HRD signature among samples with and without FA pathway gene alterations: a higher proportion of FA pathway-altered tumors exhibit a positive HRD signature (12% vs 7%, $p<0.001$). b) Concomitant alterations in other DNA repair genes within the FANCA-altered prostate cancer FMI cohort. c) In silico RNA sequencing analysis from the TCGA ovarian cancer cohort shows increased expression of the G2/M and mitotic spindle hallmarks in ovarian cancer samples with pathogenic or likely pathogenic *FANCA* genomic alterations, as compared to the *FANCA* and *BRCA1/2*-wild type control cohort. d) Heatmap illustrating individual gene expression within the mitotic spindle hallmark, including *AURKA*, in *FANCA*-altered vs wild-type TCGA tumor samples. Comparative gene expression profiles for prostate, ovarian, breast cancers, stomach, and lung adenocarcinoma are shown. *Abbreviations:* brca: breast cancer; *del: deleted; FA: Fanconi anemia pathway; HRD: homologous recombination deficiency, log2FC: log base-2 fold change; luad: lung adenocarcinoma; mut: mutant; NES: normalized enrichment score; ov: ovarian cancer; padj: adjusted p value; prad: prostate adenocarcinoma; sig: signature; stad: stomach adenocarcinoma; TCGA: The Cancer Genome Atlas; wt: wild type.*

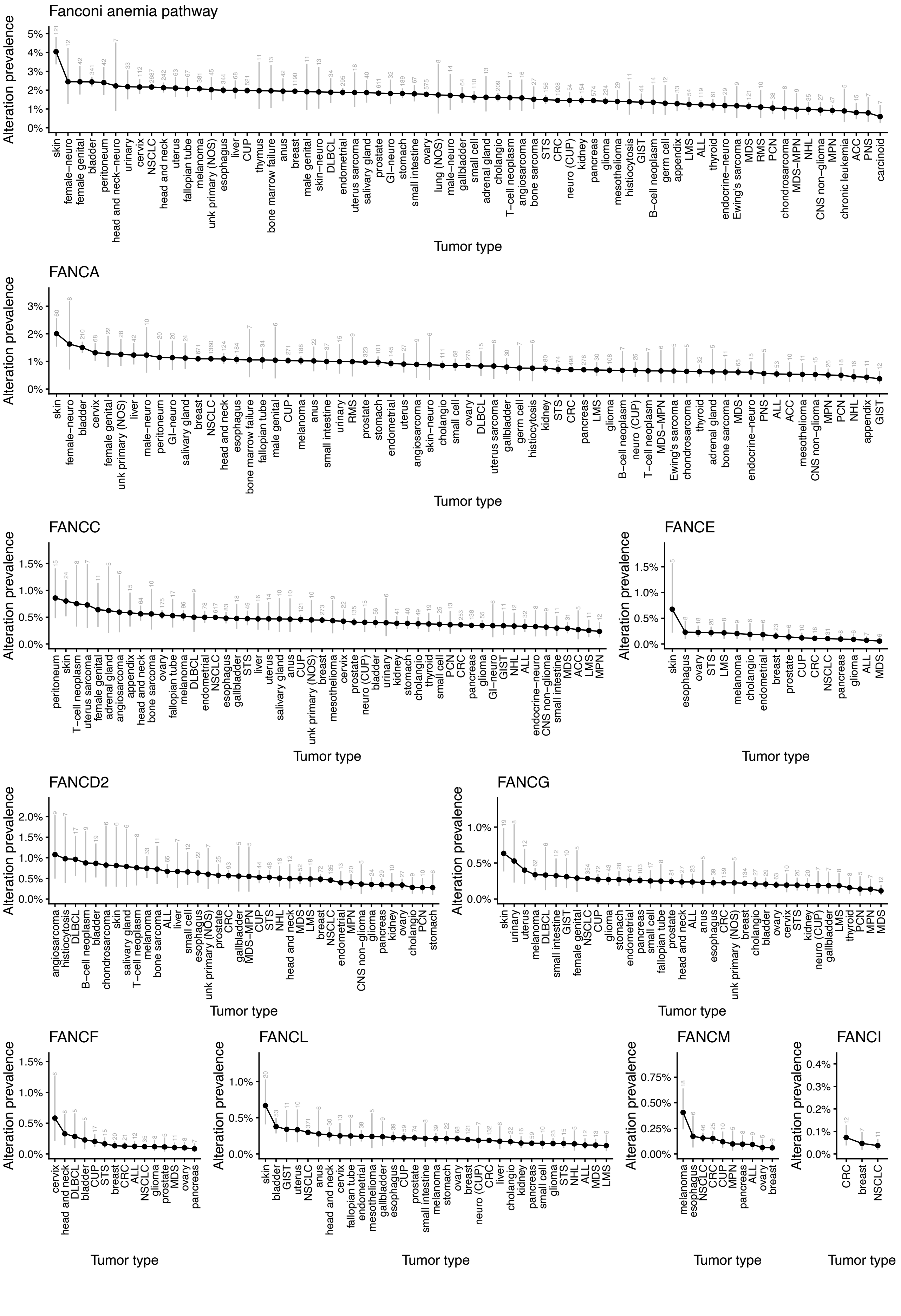

Fanconi anemia pathway
Alteration prevalence
5%
4%
3%
2%
1%
0%
skin female−neuro female genital bladder peritoneum head and neck−neuro urinary cervix NSCLC head and neck uterus fallopian tube melanoma unk primary (NOS) esophagus liver CUP thymus bone marrow failure anus breast male genital skin−neuro DLBCL endometrial uterus sarcoma salivary gland prostate GI−neuro stomach small intestine ovary lung (NOS) male−neuro gallbladder small cell adrenal gland cholangio T−cell neoplasm angiosarcoma bone sarcoma STS CRC neuro (CUP) kidney pancreas glioma mesothelioma histiocytosis GIST B−cell neoplasm germ cell appendix LMS ALL thyroid endocrine−neuro Ewing's sarcoma MDS RMS PCN chondrosarcoma MDS−MPN NHL CNS non−glioma MPN chronic leukemia ACC PNS carcinoid
Tumor type
FANCA
Alteration prevalence
3%
2%
1%
0%
skin female−neuro bladder cervix female genital unk primary (NOS) liver male−neuro peritoneum GI−neuro salivary gland breast NSCLC head and neck esophagus bone marrow failure fallopian tube male genital CUP melanoma anus small intestine urinary RMS prostate stomach endometrial uterus angiosarcoma skin−neuro cholangio small cell ovary DLBCL uterus sarcoma gallbladder germ cell histiocytosis kidney STS CRC pancreas LMS glioma B−cell neoplasm neuro (CUP) T−cell neoplasm MDS−MPN Ewing's sarcoma chondrosarcoma thyroid adrenal gland bone sarcoma MDS endocrine−neuro PNS ALL ACC mesothelioma CNS non−glioma MPN PCN NHL appendix GIST
Tumor type
FANCC
Alteration prevalence
1.5%
1.0%
0.5%
0.0%
peritoneum skin T−cell neoplasm uterus sarcoma female genital adrenal gland angiosarcoma appendix head and neck bone sarcoma ovary fallopian tube melanoma DLBCL endometrial NSCLC esophagus gallbladder STS liver uterus salivary gland anus CUP unk primary (NOS) breast mesothelioma cervix prostate neuro (CUP) bladder urinary kidney stomach cholangio thyroid small cell PCN CRC pancreas glioma GI−neuro GIST NHL ALL endocrine−neuro CNS non−glioma small intestine MDS ACC LMS MPN
Tumor type
FANCE
Alteration prevalence
1.5%
1.0%
0.5%
0.0%
skin esophagus ovary STS LMS melanoma cholangio endometrial breast prostate CUP CRC NSCLC pancreas glioma ALL MDS
Tumor type
FANCD2
Alteration prevalence
2.0%
1.5%
1.0%
0.5%
0.0%
angiosarcoma histiocytosis DLBCL B−cell neoplasm bladder chondrosarcoma skin salivary gland T−cell neoplasm melanoma bone sarcoma ALL liver small cell esophagus unk primary (NOS) prostate CRC gallbladder MDS−MPN CUP STS NHL head and neck MDS LMS breast NSCLC endometrial MPN CNS non−glioma glioma pancreas kidney ovary cholangio PCN stomach
Tumor type
FANCG
Alteration prevalence
1.0%
0.5%
0.0%
skin urinary uterus melanoma DLBCL small intestine GIST female genital NSCLC CUP glioma stomach endometrial pancreas small cell fallopian tube prostate head and neck ALL anus esophagus CRC unk primary (NOS) breast cholangio bladder ovary cervix STS kidney neuro (CUP) gallbladder LMS thyroid PCN MPN MDS
Tumor type
FANCF
Alteration prevalence
1.5%
1.0%
0.5%
0.0%
cervix head and neck DLBCL bladder CUP STS breast CRC ALL NSCLC glioma prostate MDS ovary pancreas
Tumor type
FANCL
Alteration prevalence
1.0%
0.5%
0.0%
skin bladder GIST uterus NSCLC anus head and neck cervix fallopian tube endometrial mesothelioma gallbladder esophagus CUP prostate small intestine melanoma stomach ovary breast neuro (CUP) CRC liver cholangio kidney pancreas small cell glioma STS NHL ALL MDS LMS
Tumor type
FANCM
Alteration prevalence
0.75%
0.50%
0.25%
0.00%
melanoma esophagus NSCLC CRC CUP MPN pancreas ALL ovary breast
Tumor type
FANCI
Alteration prevalence
0.4%
0.3%
0.2%
0.1%
0.0%
CRC breast NSCLC
Tumor type

Supplementary Figure 10. Frequencies by organ/tumor site for FA pathway gene alterations including distribution for individual gene alterations are illustrated. Frequencies by organ/tumor for whole FA pathway genes group, as well as individually for *FANCA, FANCC, FANCE, FANCD2, FANCG, FANCF, FANCL, FANCM and FANCI* genes are illustrated. *Abbreviations: ACC: adenoid cystic carcinoma; ALL: acute lymphoblastic leukemia; CRC: colorectal cancer; CUP: cancer of unknown primary; DLBCL: diffuse large B-cell lymphoma; FA: Fanconi anemia; GIST: gastrointestinal stromal tumor; LMS: leiomyosarcoma; MDS: myelodysplastic syndrome; MPN: myeloproliferative neoplasm; NHL: non-hodgkin lymphoma; NOS: not otherwise specified; NSCLC: non-small cell lung cancer; PCN: pancreatic cystic neoplasm; PNS: peripheral nervous system; RMS: rhabdomyosarcoma; STS: soft tissue sarcoma.*

A

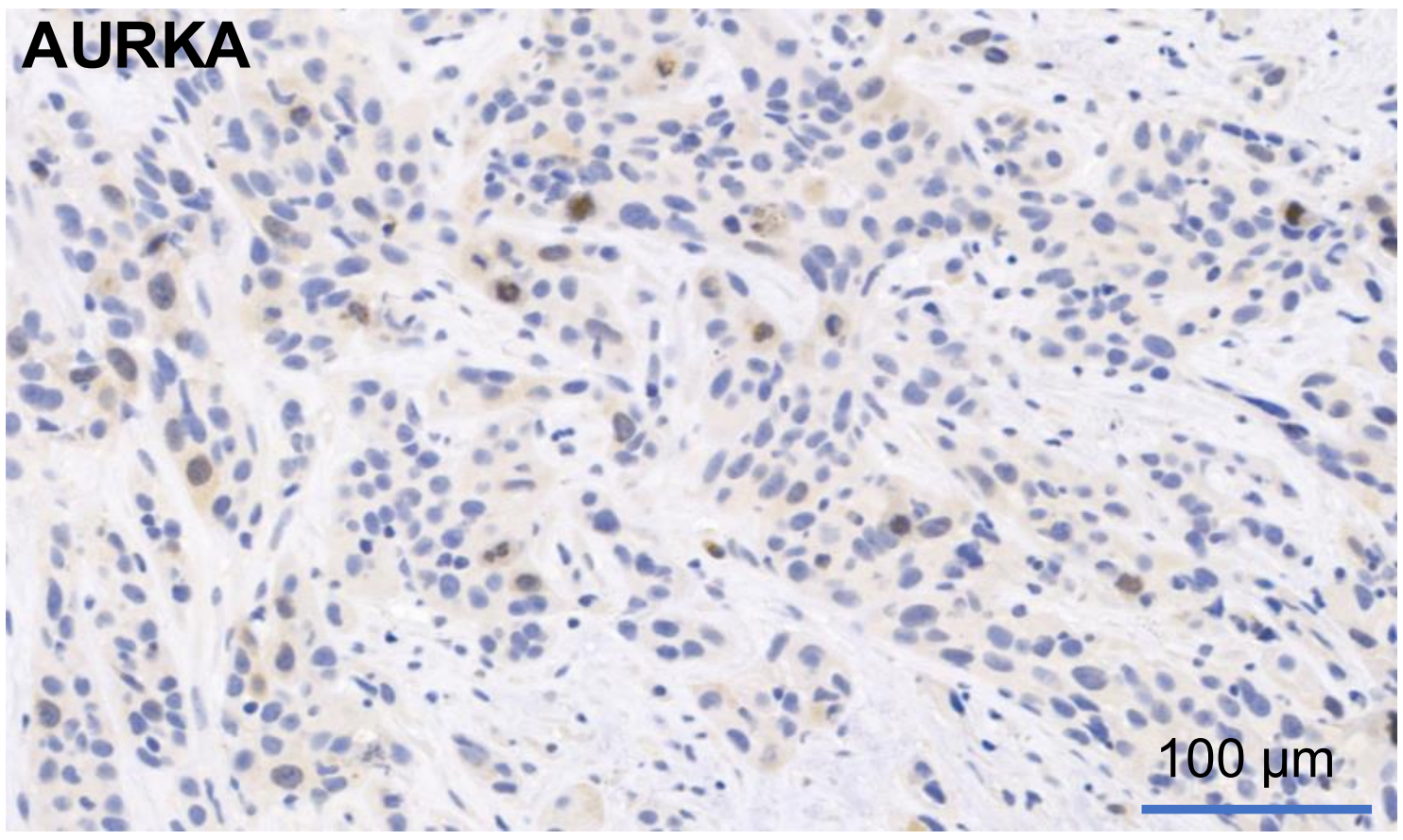

B

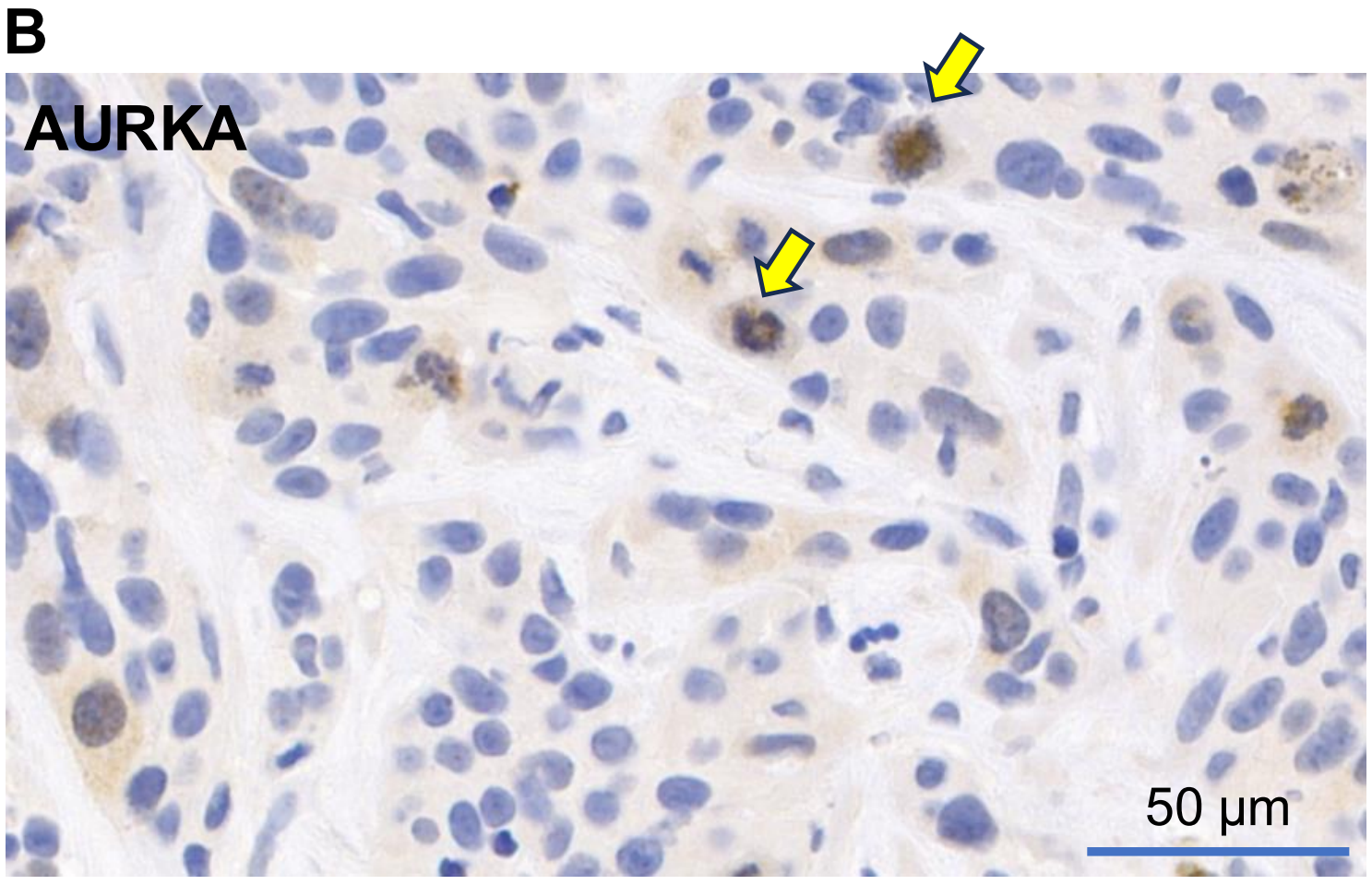

Supplementary ementary Figure 11. AURKA IHC staining on a head-and-neck squamous *FANCA*-mutated (p.R880* (c.2638C>T), VAF 30%) carcinoma FFPE sample. a-b). Diffuse, weak cytoplasmic and occasional nuclear staining (mean H-score nuclear/cytoplasmic combined: 33), as well as specific moderate to strong centrosomal staining in mitotic cells (yellow arrows, b), is observed. *Abbreviations: AURKA: Aurora kinase A; FFPE: formalin-fixed paraffin-embedded; μM: micromolar; VAF: variant allele frequency.*

A

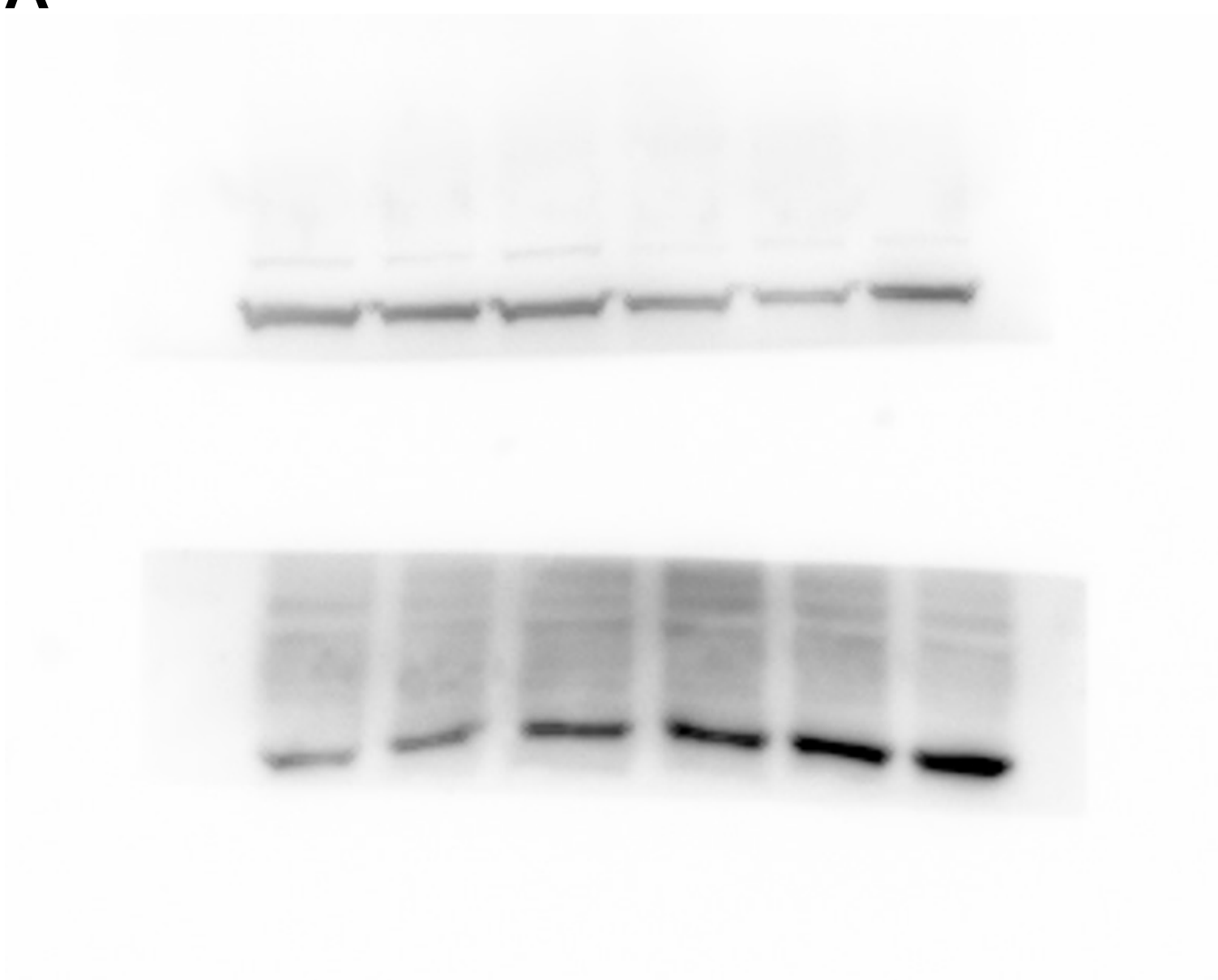

B

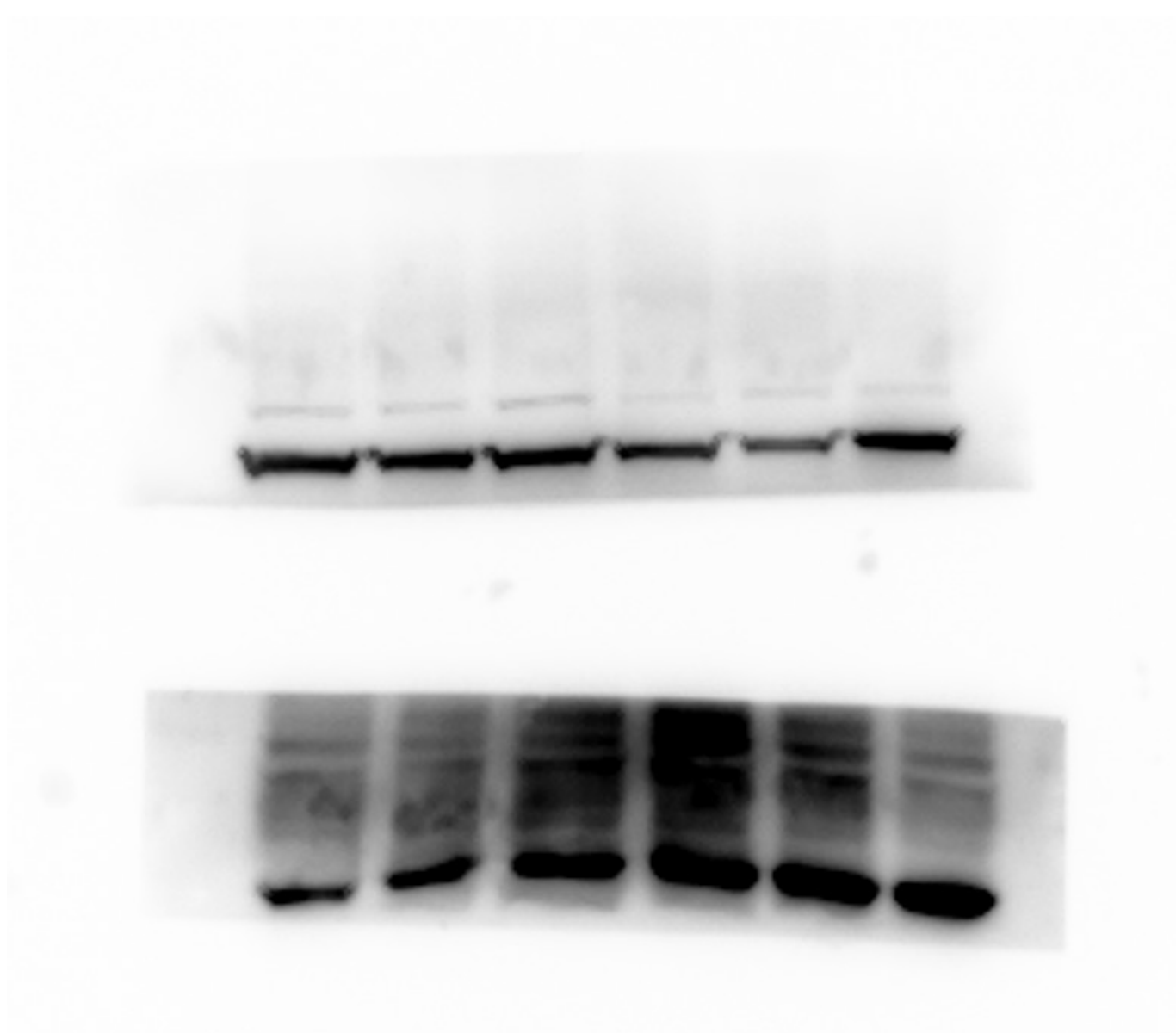

Supplementary ementary Figure 12. Original western blot corresponding to Figure 2F. a-b) Original blot used to crop AURKA (a) and Vinculin (b). *Abbreviations: AURKA: Aurora kinase A.*

A

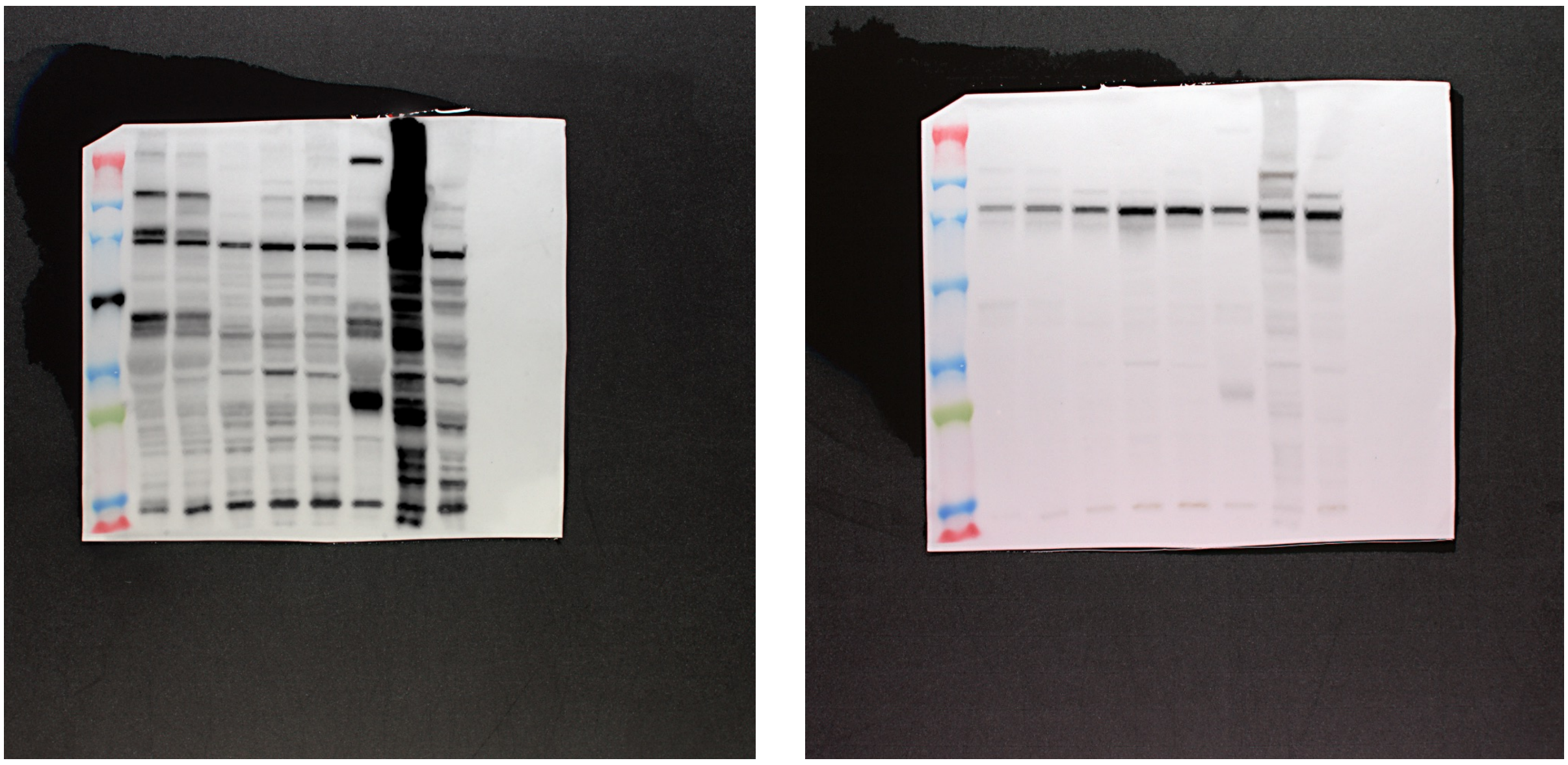

B

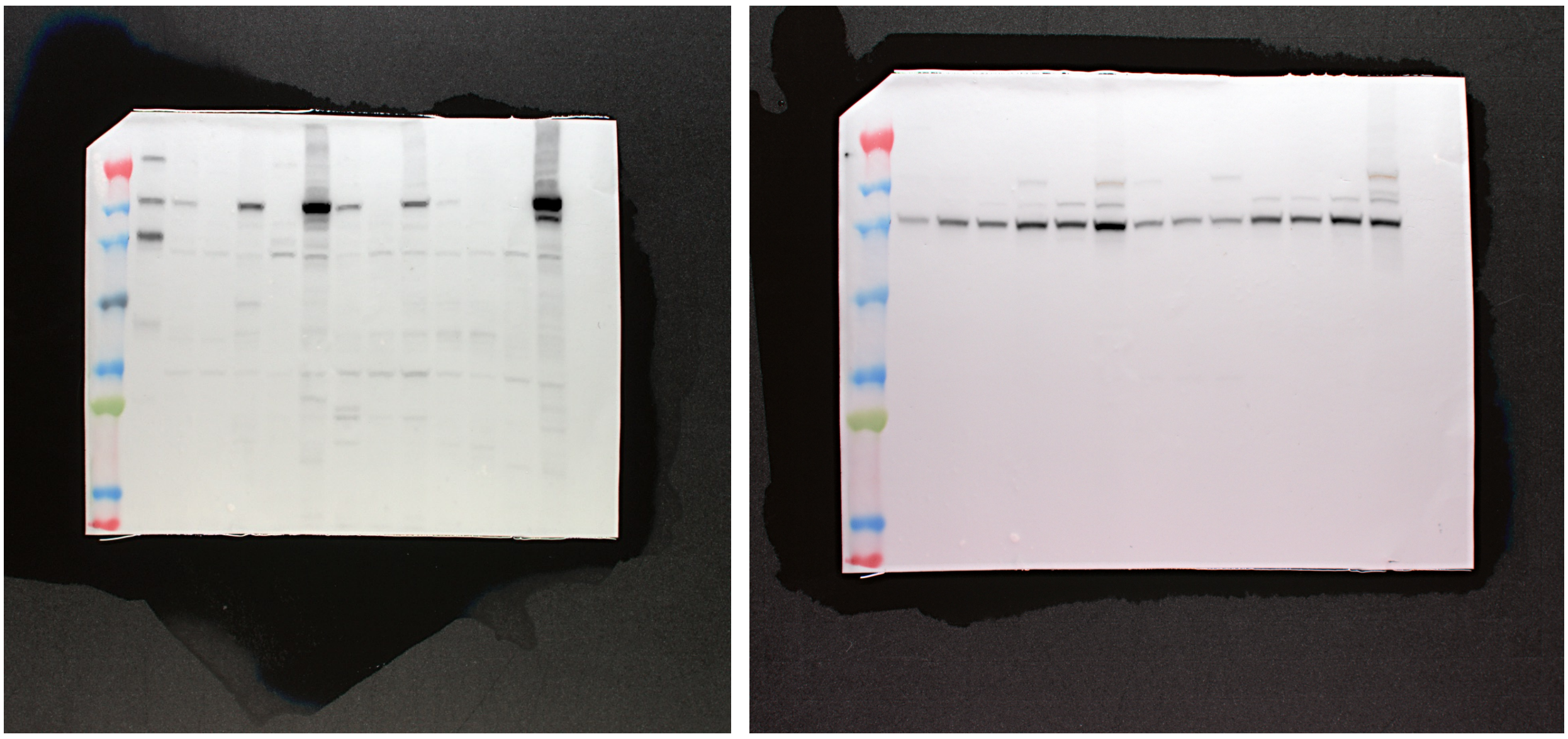

Supplementary ementary Figure 13. Original western blot corresponding to Supplementary ementary Figure 3A. a) Original blot used to crop FANCA and Vinculin for the left part of the figure (DU145 cells). b) Original blot used to crop FANCA and Vinculin for the right part of the figure (CCH-SCC-FA1 and UM-SCC-01 cells).

A

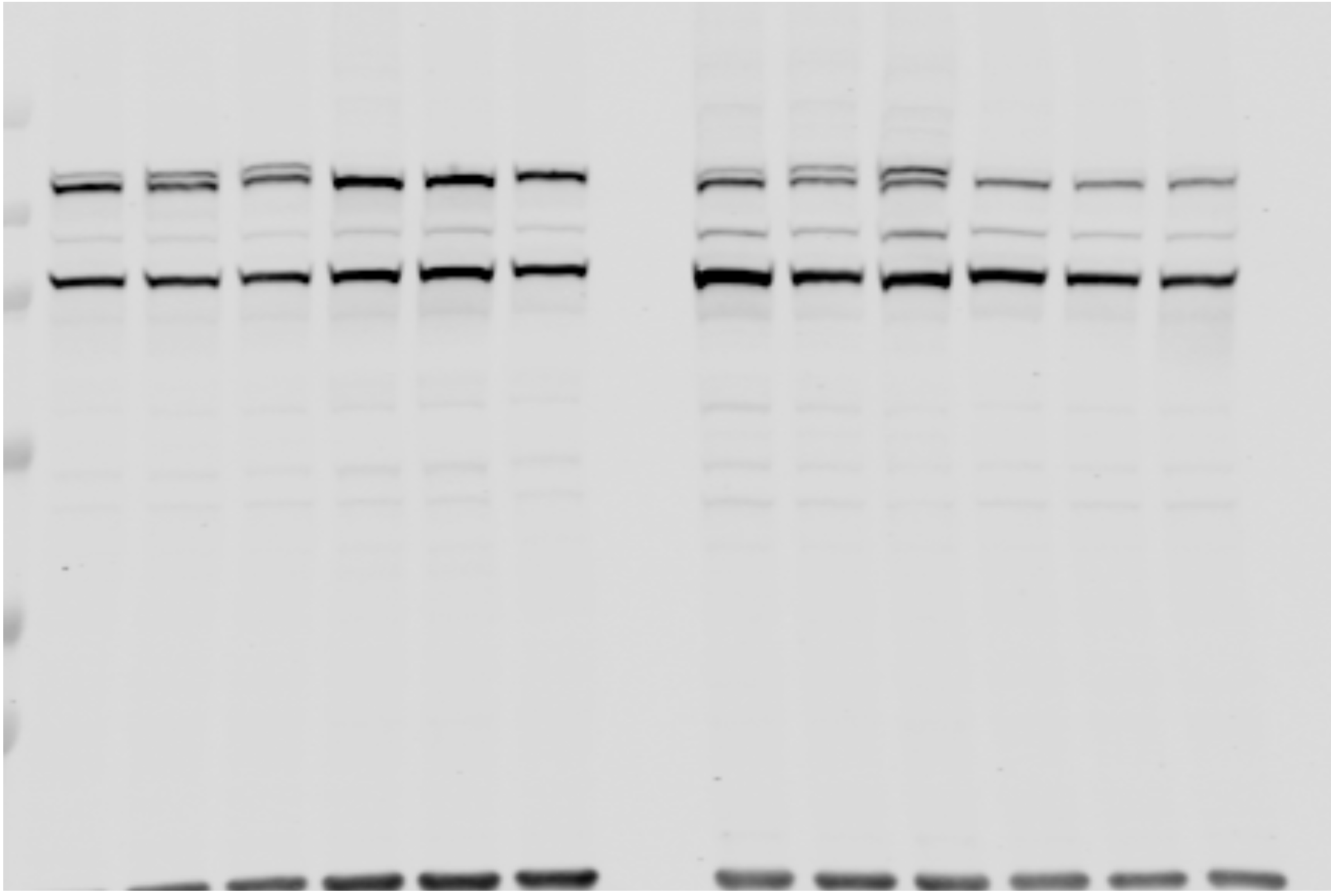

Supplementary ementary Figure 14. Original western blot corresponding to Supplementary ementary Figure 3B and 3C. a-b) Original blot used to crop FANCD2 and Vinculin for the DU145 and CCH-SCC-FA1 cells.
